# A Phylogenomic Framework and Divergence History of Cephalochordata Amphioxus

**DOI:** 10.3389/fphys.2018.01833

**Published:** 2018-12-18

**Authors:** Qi-Lin Zhang, Guan-Ling Zhang, Ming-Long Yuan, Zhi-Xiang Dong, Hong-Wei Li, Jun Guo, Feng Wang, Xian-Yu Deng, Jun-Yuan Chen, Lian-Bing Lin

**Affiliations:** ^1^Faculty of Life Science and Technology, Kunming University of Science and Technology, Kunming, China; ^2^Evo-Devo Institute, School of Life Sciences, Nanjing University, Nanjing, China; ^3^State Key Laboratory of Grassland Agro-Ecosystems, College of Pastoral Agricultural Science and Technology, Lanzhou University, Lanzhou, China; ^4^State Key Laboratory of Palaeobiology and Stratigraphy (LPS), Nanjing Institute of Geology and Palaeontology, Chinese Academy of Sciences, Nanjing, China

**Keywords:** Cephalochordata, amphioxus, phylogenomics, divergence history, speciation process, protein domains, evolutionary genomics

## Abstract

Amphioxus, or cephalochordates, are often used as the living invertebrate proxy of vertebrate ancestors and are widely used as evolutionary biology models of chordates. However, their phylogeny, divergence history, and speciation characteristics remain poorly understood, and phylogenomic studies to explore these problems lacking entirely from the literature. Here, we determined a new transcriptome of *Branchiostoma japonicum*. Combined with mass sequences of all other 18 species, a 19-way phylogeny was constructed via multiple methods (ML, BI, PhyloBayes, and ASTRAL), consistently supporting a phylogeny of [(*B. belcheri* + *B. japonicum*) + (*B. lanceolatum* + *B. floridae*) + *Asymmetron lucayanum*] in amphioxus. Congruent phylogenetic signals were found across mitochondrial genes, 12S RNA, and complete mitochondrial genomes according to previous reports, indicating that 12S RNA may have potential as a molecular marker for phylogenetic analysis in amphioxus. Molecular dating analysis indicated a radiation of the cephalochordates during the Cretaceous (∼104-61 million years ago), supporting an association between the diversification and speciation of cephalochordates with continental drift and associated changes in their respective habitats during this time. The identified functional enrichment analysis for species-specific domains indicated that their function mainly involves immune response, apoptosis, and lipid metabolism and utilization, signaling that pathogens and changes of energy requirements are an important driving force for amphioxus speciation. This study represents the first large-scale phylogenomic analysis of most major amphioxus genera based on phylogenomic data, providing a new perspective on both phylogeny and divergence speciation of cephalochordates.

## Introduction

Amphioxus, also known as lancelets, belongs to Leptocardii: Amphioxiformes and is the only modern representative of the subphylum Cephalochordata (Koop and Holland, 2008). Amphioxus is a key topic for investigations of evolutionary biology as they provide indications about the evolutionary origins of vertebrates, and provide an intriguing comparison point to trace how vertebrates have evolved and adapted (Shimeld and Holland, 2005; Putnam et al., 2008). Despite the separate evolution of cephalochordate and vertebrates from a common ancestor more than 520 million years ago (Mya), their body structure and morphology maximally retained characteristics of these vertebrate ancestors (*Haikouella lanceolata*) (Chen et al., 1999). With the recent accumulation of genetic data on amphioxus, the molecular phylogeny, evolutionary developmental biology (evo–devo), and genomics further suggest amphioxus as a valid model of vertebrate ancestry (Shimeld and Holland, 2005; Putnam et al., 2008). The amphioxus of today is the only living cephalochordates and is distributed in three genera: *Branchiostoma* (∼28 species) with the largest number, *Epigonichthys* (one species), and *Asymmetron* (one species). However, additional cryptic species might exist (Yue et al., 2014). *Branchiostoma* widely distributed in the mid-low latitudes of the Atlantic Ocean, the Mediterranean, and the Pacific Ocean, with the remaining two genera primarily distributed in the mid-low latitudes and tropics along the equatorial zone. The habitat of amphioxus can be found in shallow coastal seawater at a depth of 8–16 m, and the animals hide most of their bodies in the fine sand of the seafloor, finding their food via filter-feeding of the seawater.

Despite the important role of amphioxus in evolutionary biology, publications investigating the molecular phylogeny within amphioxus are few, especially in recent years. In contrast, many studies focus on scientific questions involving evolutionary biology and molecular phylogeny between amphioxus and vertebrates, which leads to a bias in our knowledge of amphioxus evolution. Previous studies of the last decade focused on amphioxus taxonomy and species divergence based on mitochondrial genomics (mitochondrial DNA, mtDNA). For example, Luo et al. (2007) used the cytochrome c oxidase subunit 1 (*cox1*) and cytochrome b subunit (*cob*) gene fragments to construct phylogenetic trees among *Branchiostoma belcheri, B. japonicum*, and *B. tsingtaoensis*, and calculated average genetic distance among them. The results showed a clustered clade and intraspecific differences between *B. japonicum* and *B. tsingtaoensis*, supporting the classification of *B. tsingtaoensis* as *B. japonicum*. Zhong et al. (2009) sequenced the complete mtDNA of both *B. japonicum* and *B. belcheri* and combined the results with the reported 13 protein-coding genes (PCGs) of mtDNA from five other species of amphioxus to construct a maximum likelihood (ML) tree of protein and nucleic acid sequences, respectively. Both phylogenetic trees supported the results of Luo et al. (2007); moreover, the results of these investigations indicated that amphioxus living in Beihai, Guangxi province and Maoming, Guangdong province belongs to the same species *B. belcheri* (Zhong et al., 2009). Furthermore, Zhong et al. (2009) further constructed a phylogenetic tree containing different geographical populations of amphioxus based on the 12S rRNA (ribosomal RNA) sequence of mtDNA. It is worth noting that the tree constructed by 13 PCGs supported the following topological structure: ((((*B. belcheri* + *B. japonicum*) + *Branchiostoma lanceolatum*) + *Branchiostoma floridae*) + *Asymmetron*), while the 12S rRNA-based tree did not support *B. lanceolatum* as a sister lineage of the (*B. belcheri* + *B. japonicum*) clade instead of *B. floridae*, namely [(*B. belcheri* + *B. japonicum*) + (*B. lanceolatum* + *B. floridae*) + *Asymmetron*]. This inconsistency may be caused by limited sequence site information and use of improper molecular markers (Zhong et al., 2009). Kon et al. (2007) found the new species of amphioxus *Asymmetron inferum*, near whale corpses on the seafloor at a depth of 229m (Nommisaki cape, southwest of Kyushu island, Japan) and used the complete mtDNA of eight amphioxus species (including three other species as outgroups) to reconstruct their Bayesian (BI), ML, and maximal parsimony (MP) trees. The topology was consistent with that constructed via 12S rRNA, rather than that based on 13 PCGs by Zhong et al. (2009). The phylogenetic relationship among these four *Branchiostoma* species thus remained unclear and this divergence caused by various mtDNA genes still requires clarification.

Molecular dating based on phylogenetic tree was widely investigated within amphioxus species. Kon et al. (2007) estimated the divergence time of eight amphioxus species using molecular dating methods, showing that *Asymmetron* and *Branchiostoma* genera split from their common ancestor (*Asymmetron* – *Branchiostoma*) about 240 Mya, and *B. belcheri* – *B. floridae* about 130 Mya. However, more recently, Yue et al. (2014) constructed a ML tree of 15 species based on 427 orthologs using transcriptomic and genomic data and including two amphioxus species (*B. floridae* and *A. lucayanum*). They suggested a divergence time of *Asymmetron* – *Branchiostoma* of about 120 Mya. Huang et al. (2014) performed genome sequencing of *B. belcheri* (reporting the second amphioxus genome after that of *B. floridae*) and estimated the divergence times of 15 species based on 513 orthologs from the genomic sets; their data covered the two amphioxus species, *B. floridae* and *B. belcheri*, which predicted 130 Mya as the divergence time between them. So far, phylogenetic trees containing multiple amphioxus species have been investigated based on mtDNA; however, those recently constructed by large-scale transcriptomic and genomic data contained only two amphioxus species (no more than two species). Therefore, it is necessary to reconstruct a phylogenetic tree containing multiple amphioxus species and to calculate the divergence time frame at the omics level to further uncover the divergence history and to alleviate the contradictions among previous investigations.

Additionally, previous investigations performed a direct comparison of the domain diversity between amphioxus and other model vertebrates, identifying amphioxus-specific protein domains from *B. belcheri* and *B. floridae* (Huang et al., 2014). Nevertheless, lineage-specific domains among multiple amphioxus species and their biological function remain largely unknown, which obstructs our understanding of the characteristics of amphioxus speciation.

Here, we sequenced the transcriptome of adult *B. japonicum*, and assembled, respectively, it and *A. Lucayanum* transcriptome. Combined with the whole-genome gene set for the other 16 species and the transcriptome of *B. lanceolatum*, we performed large-scale comparative analyses to reconstruct the phylogenetic tree and estimate divergence times among cephalochordates. Moreover, species-specific and ancestral domains were identified among amphioxus and functional enrichment analyses were performed for each target domain set.

## Materials and Methods

### Ethics Statement

This study was carried out in accordance with the recommendations of the Guide for the Care and Use of Laboratory Invertebrate Animals. The protocol was approved by the Ethical Committee of Researches of the Nanjing University (NJU).

### Materials and RNA Sequencing

Adult species of *B. japonicum* were collected from Qingdao, Shandong province, China, and kept in laboratory for 5 days to facilitate emptying of the digestive tract. Total RNA was isolated from several male and females using the TRIzol reagent (Invitrogen, United States), and genomic DNA was removed by RNase-Free DNase Set (Qiagen, Germany) according to the manufacturer’s instructions. RNA quality was determined using Bioanalyser 2100 (Agilent, United States), and the concentration was measured using the NanoDrop 1000 (Thermo Scientific, United States). Standard cDNA libraries were constructed using standard Illumina libraries prep protocols and TruSeq kit (Illumina, United States). After homogenization treatment of sequencing libraries, RNA sequencing (RNA-seq) was conducted on an Illumina HiSeq^TM^ 2000 with 100-bp paired-end reads. RNA-seq was performed in BGI (Shenzhen, China).

### Assembly of Reads, Correction of Gene Models, and Functional Annotation

We firstly used FastQC (Brown et al., 2017) to control the quality of the raw sequencing reads by checking for over-represented and potentially contaminant sequences following stringent criteria: (1) reads with adaptors were discarded; (2) reads with unknown bases > 10% were discarded; (3) reads with a length < 20 bp were discarded. We used the program Sickle to remove or trim low-quality reads [percentage of low-quality bases (bases with sequencing quality score ≤ 5) > 50%] (Cabili et al., 2011). Quality paired-end reads (clean reads) were obtained and then used to assemble the transcriptome using Trinity (Grabherr et al., 2011). In addition, we obtained clean reads of a further adult amphioxus (*A. lucayanum*) (Accession: SRX437621) from the Sequence Read Archive (SRA) database of the NCBI, and assembled its transcriptome following the above pipelines. Trinity can detect potential isoforms from alternative splicing and label them with the same prefix. For multiple isoforms, the longest unigene was selected from each isoform group as a unique representation for that group. We also downloaded the *B. lanceolatum* transcriptome from the NCBI Transcriptome Shotgun Assembly (TSA) database (Accession numbers: JT846176-JT905674) (Silvan et al., 2012). Potential coding sequences (CDSs) of three amphioxus species were predicted via Blastx (default parameter) (Mount, 2007) search of the NCBI non-redundant (NR) protein database. The optimal alignment was extracted as template for the determination of the CDSs of unigenes. Then, ESTScan (*E*-value = 10^-5^) (Iseli et al., 1999) software was used to predict the CDS of unigenes that failed to produce any hit.

The protein, mRNA, and CDS sequences of 16 sampled species with sequenced genome were retrieved from online databases (see **Supplementary Table S1** for details). Among these genomic sequences, the CDS and protein sequence of 15 species (except for *A. mississippiensis*) could not be completely matched, thus they were corrected. First, CDS sequences below 120 bp were removed; then, protein sequences were aligned to mRNA using Exonerate software (Guy and Birney, 2005). Based on amino acid-nucleic acid alignments, incompatible CDS and protein sequences were corrected, discarding uncorrected sequences. For the protein sequences translated from corrected CDSs, we used the Blastp tool to search for them in Nr, Swiss-Prot, and gene ontology (GO) databases (**Table 1**), respectively, performing functional annotation of all genes or unigenes.

**Table 1:** Summary statistics for sequencing, assembly, and annotation of transcriptomes.

	*B. japonicum*	*A. lucayanum*
Raw reads	∼52 million	∼146 million
Clean reads	∼49 million	∼135 million
Q20 percentage	97.09%	/
N50 (bp)	1,753	1,797
Number of final unigenes	92,003	112,753
Number of CDSs	50,316	42,148
NR	46,540	/
Swiss-Prot	31,941	/
GO	30,319	/

### Construction of Orthologous Alignments

HaMSTR (Ebersberger et al., 2009) and reciprocal best hits (BRH) (Moreno-Hagelsieb and Latimer, 2008) methods are generally used for the construction of orthologous genes. The results obtained from HaMSTR and BRH methods were intersected as a final ortholog set of 19 sampled species, to avoid bias caused by using a single method only. For the HaMSTR approach, according to previous description in transcriptomic analyses (Misof et al., 2014; Li et al., 2015), a set of core-orthologs was constructed from nine vertebrate genomes including human (*Homo sapiens*), mouse (*Mus musculus*), chicken (*Gallus gallus*), zebrafish (*Danio rerio*), Coelacanth (*Latimeria chalumnae*), fugu (*Takifugu rubripes*), elephant shark (*Callorhinchus milii*), lamprey (*Petromyzon marinus*), and Western clawed frog (*Xenopus tropicalis*). All 6,793 one-to-one core-orthologous proteins were obtained from the OrthDB v9.1 database (Kriventseva et al., 2015) and were inputted into the multiple alignment tool Muscle (v3.8) (Edgar, 2004) using default sets. Then, a hidden Markov model “primer taxa” was built from the multiple alignment of the core-orthologs using the hmmbuild tool of the HMMER3 package (Finn et al., 2011). The “primer taxa” served as input to produce the core-ortholog database for the program HaMStR v.13.2.6 (Ebersberger et al., 2009) to search for the corresponding orthologs in the remaining 10 species. If one species contained multiple corresponding orthologs in co-orthologous, only the optimal hit was retained. HaMStR v.13.2.3 was run using strict parameters (-sequence file, -est, -hmmset, -refspec, -representative, and -ublast) according to a previous description (Li et al., 2015). Furthermore, Proteinortho (v5.13) (*E*-value = 10^-10^) (Lechner et al., 2011), software compiled based on the BRH method that is widely used for large-scale comparative genomic analysis, was employed to identify co-orthologous among all 19 species. The PhyloTreePruner pipeline was employed to pick the unique representative for each species in co-orthologous based on the gene tree (Kocot et al., 2013). According to the gene labels, the obtained orthologous gene groups were collectively included in the results from HaMSTR and BRH methods. All orthologous genes, including protein and CDS sequences, were aligned using the Prank tool (“-codon” option) (Löytynoja and Goldman, 2005), and then further trimmed via Gblocks (Talavera and Castresana, 2007) with the parameter “-t = c” to remove poorly aligned regions. Trimmed alignments that contained shorter than 60 bp/20 amino acids were removed.

### Phylogenetic Analysis

Concatenated alignments used for the construction of the phylogenetic tree were constructed from all orthologs using the FasParser package (Sun, 2017). The best amino acid substitution model was identified for the further phylogenetic analysis using ProtTest (v3.4) (Abascal et al., 2005). The LG+I+G+F model was recommended as the best model based on both the Akaike information criterion (AIC) and Bayesian information criterion (BIC). Phylogenetic analyses were conducted for all aligned protein dataset using site-homogeneous (ML and Bayes) and -heterogeneous models (PhyloBayes). RAxML 7.0.4 (Guindon et al., 2010) was used to constructed ML trees, and 1000 bootstraps (BS) were used to estimate node reliability. The Bayesian phylogenetic tree was reconstructed using MrBayes 3.2.2 (Ronquist et al., 2012). In MrBayes analysis, the LG model was not available; thus, the JTT+I+G+F model was used as the secondary best choice (Yue et al., 2014). Two independent runs with four chains (three heated and one cold) were conducted concurrently for 1,000,000 generations, sampling every 100 generations. When the estimated sample size (ESS) value exceeded 100 and the potential scale reduction factor (PSRF) was close to 1.0, stationarity was considered to be reached, as recommended by the MrBayes software (Ronquist et al., 2012). Next, the first 25% samples were discarded as burn-in, and the branch lengths and posterior probabilities (PP) were calculated in a consensus tree. Bayesian analyses with a site-heterogeneous model were implemented using PhyloBayes 4.1b (Lartillot et al., 2013). After the removal of constant sites from the alignment, two independent chains, starting from a random tree, were run under the CAT-GTR model.

Next, we also used ASTRAL II, a statistically consistent algorithm to estimate the species tree topology under the multi-species coalescent model (Mirarab et al., 2016). Support values were obtained by calculating the local posterior probability, a advantage of ASTRAL II that presented high precision compared to multi-locus bootstrapping on a wide set of simulated and biological datasets (Sayyari and Mirarab, 2016). We conducted the analysis without the “species map” set, which means that it is unnecessary to assign multiple individuals to the same species to one taxon. Species tree obtained from RAxML gene trees of each of the 3070 orthologous loci, summarized with ASTRAL II. As species tree analyses do not require outgroups (Heled and Drummond, 2009), all 19 species thus were included as ingroups in the ASTRAL II analyses.

### Estimation of Divergence Time

Based on the ML tree reconstructed above, Bayesian estimation was performed for the divergence time of the sampled 19 species to construct a time frame of cephalochordate evolution. The nine divergence nodes were signed as calibration points according to previously summarized fossil records (Benton and Donoghue, 2007; Yue et al., 2014). A calibration point (520.00–? Mya) from our early research was used at Olfactores (n) (Chen et al., 1999). In molecular dating analysis, a safe upper bound for the root age is essential in Markov chain Monte Carlo Tree (MCMCTree). Since no reliable fossil record exists that could be used here, the secondary calibration approach was used to obtain the root age (q, Deuterostomia). Currently, the divergence time at the Deuterostomia node was estimated based on the tree with the root node of Bilateria (Deuterostomia+Mollusca) via molecular clock. However, various values have been reported for the maximum constraints of the crown bilaterian divergence time, including 581.5 Mya (Benton and Donoghue, 2007), and 640–730 Mya (Peterson et al., 2008). Yue et al. (2014) explored the divergence time of crown bilaterian via MCMCTree analysis, including well-described fossil calibrations, under fixed root lower bounds (531.50) and a variety of upper bounds (600, 700, 800, and 900 Mya). Then, the authors proposed that 600 and 700 Mya were reliable and calculated their respective divergence time of Deuterostomia based on both calibration constraints (resulting in 532.67–598.27 and 585.50–698.50 Mya) (Yue et al., 2014). Consequently, we employed both results as different secondary calibration dates for the Deuterostomia (q) node in the current investigations. All calibration constraints of divergence nodes are presented in **Table 2**.

**Table 2:** Bayesian MCMC and R8S estimations for the divergence time of each internal node shown in **Figure 2**, assuming that the Deuterostomia divergence (root calibration constraints) occurred at [532.67, 598.27] Mya.

		Calibration constraints	MCMCTREE		R8S	
Node index	Node name	[min, max] (Mya)	Mean (Mya)	95% CI (Mya)	Mean (Mya)	95% CI (Mya)
a	Eutheria	[61.50, 100.50]	76.49	[60.91–99.43]	71.65	[56.72–97.68]
b	Amniote	[312.30, 330.40]	318.63	[312.00–329.34]	312.67	[301.47–326.36]
c	Ankylopoda	[259.70, 299.80]	268.28	[258.33–291.27]	257.36	[253.21–284.63]
d	Archosauriformes	[235.00, 250.40]	244.31	[235.42–250.63]	246.28	[237.34–249.69]
e	Tetrapoda	[330.40, 350.10]	343.13	[331.80–350.42]	344.07	[331.78–351.51]
f	Sarcopterygii	–	382.15	[354.82–407.11]	367.22	[333.78–398.61]
g	Osteichthyes	[416.00, 421.75]	418.68	[416.02–421.73]	424.82	[418.13–431.44]
h	Acanthopterygii	[149.85, 165.20]	157.36	[149.81–165.20]	152.92	[146.65–166.62]
i	Gnathostomata	[412.75, 462.50]	443.11	[420.23–461.68]	448.65	[413.27–477.76]
j	Vertebreta	[460.60, –]	494.47	[460.94–532.75]	471.19	[452.53–498.87]
k	Olfactores	[520.00, –]	548.41	[513.66–579.60]	525.77	[504.49–546.35]
l	Chordata	–	577.52	[544.32–597.51]	546.06	[502.15–588.26]
m	–	–	61.11	[9.93–128.65]	64.23	[12.58–123.75]
n	Branchiostomidae	–	86.83	[39.98–149.47]	91.11	[32.84–162.85]
o	–	–	72.42	[30.50–140.35]	78.64	[34.22–143.95]
p	Cephalochordata	–	104.37	[21.25–191.35]	112.09	[28.80–193.76]
q	Deuterostomia	[532.67, 598.27]	587.14	[557.15–601.25]	569.53	[543.25–589.17]
r	Ambulacraria	–	569.65	[461.45–629.35]	547.27	[414.91–601.75]

Molecular dating analysis was conducted using the MCMCTree subprogram of the PAML package (v4.8) with the concatenated gene matrix used in the phylogenetic analysis (Dos and Yang, 2011). As fixed topology from the guide tree, we first obtained the ML estimates of the branch lengths, the gradient (G) vector, and the Hessian (H) matrix using the codeml from the PAML package (v4.8) (Dos and Yang, 2011), then, these estimated values were fed into control files of MCMCTree together with the LG substitution model. We set a time unit of 1 million years, and used the same gamma prior G(1, 100) to specify the substitution rate and rate drift parameters. An autocorrelated and lognormal relaxed clock model (Rannala and Yang, 2007) was used to estimate the posterior distribution of the divergence time, given these priors. The burn-in for the MCMC chain was run 10,000,000 generations, with sampling parameters every 1,000 generations. Two independent runs were conducted to ensure convergence of MCMC chains. The program summarized the mean and the 95% confidence intervals (CIs).

The penalized-likelihood (PL) approach was used in the program r8s V1.8 (Sanderson, 2003), in combination with the truncated-Newton algorithm (Sanderson, 2002). The cross-validation method in r8s was used to determine the optimal level of rate-smoothing of the PL analyses with smoothing parameters varying from 1 to 1,000 according to a previously described procedure (Mulcahy et al., 2012). The optimal smoothing parameter (*S* = 100) was first determined using the same fixed topology (ML) used as input for MCMCTree, since there is evidence that branch lengths are more accurately estimated by ML than by BI methods (Schwartz and Mueller, 2010). CIs for the PL age estimates were obtained by replicating PL analyses of 1,000 trees. The mean divergence time and 95% CIs were summarized using the r8s bootstrap kit.

### Analysis of Molecular Evolution

**FIGURE 1:**
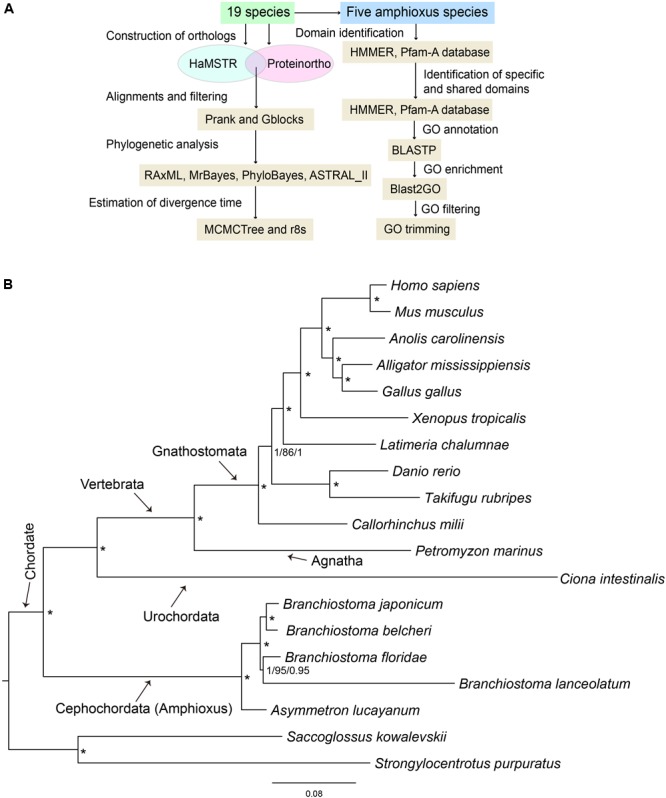
**(A)** Pipeline of primary software used in this study. **(B)** 19-way maximum-likelihood (ML) tree inferred from the concatenated 397 orthologous gene groups. The branch length is proportional to the expected amino acid substitution rate and the scale bar represents 0.08 expected amino acid substitutions per site. Numbers on the internal nodes represent Bayesian posterior probabilities (PP1, left) of MrBayes, bootstrap values (BS, middle) of RAxML, and Bayesian posterior probabilities (PP2, right) of PhyloBayes. An asterisk indicates PP1 = 1.0, BS = 100, and PP2 = 1.0. The Bayesian and PhyloBayes trees are shown in **Supplementary Figures S1, S2**, respectively.

To identify the domains contained in the protein sequences for all amphioxus, the amino acid sequence sets of five species of amphioxus were searched in Pfam-A database 31.0 using local HMMER 3.1^1^. This is the best program for domain identification and it has often been used together with a profile database, such as Pfam (Rekapalli et al., 2009). The domain ID set of each amphioxus was fed into a Venn Diagram^2^ to obtain specific and shared domain types among the five investigated species of amphioxus. It is worth pointing out that only *B. belcheri* and *B. floridae* protein sets were generated from the sequenced whole genome, while those of the other three species were obtained from *de novo* assembled adult transcriptomes that contained assembly defects and missing protein sequences. However, a domain type could be contained in a variety of protein sequences and the domain size was short (generally in the range of 40–50 amino acids), which effectively avoided bias caused by incomplete species protein sets and *de novo* assembly errors. Domain sequences were annotated to GO databases using the Blastp tool with default *E*-value. GO enrichment analysis for specific and shared domains among all amphioxus was performed via Fisher’s exact test, that was implemented in the Blast2GO pipeline (Conesa et al., 2005). The Benjamini-Hochberg (BH) method was used for false discovery rate (FDR) correction for the Fisher test (FDR threshold values = 0.05). The list of significant GO terms was further filtered using GO trimming (v2.0) (Jantzen et al., 2011) to discard redundant terms. Pipeline of primary software used in this study was presented in **Figure 1A**.

## Results

### Sequencing, Assembly, and Annotation

Illumina sequencing for *B. japonicum* generated ∼52 million raw reads. After quality control for the original data, ∼49 million clean reads were obtained, and the Q20 percentage reached 97.09%, indicating a high quality of transcriptome sequencing (**Table 1**). Clean data have been submitted to the NCBI SRA database: Accession number SRX448311. By Trinity initial assembly, we obtained 161,542 contigs with an N50 value of 816 bp and an average length of 375 bp. Further overlapping clustering for contig sequences, we finally obtained 92,003 unigenes with an N50 value of 1,753 bp and an average length of 785 bp. The assembly results of *A. lucayanum* were nearly consistent with previous reports that originally sequenced its transcriptomes (Yue et al., 2014). Furthermore, we aligned pair-end reads back to unigene sequences of *B. japonicum*, 86.15% clean reads could be completely matched, indicating that the assembly quality meets requirements for subsequent analysis. Based on unigene set obtained, 46,145 and 4,171 CDS sequences were obtained by homologous alignment search and ESTScan detection, respectively. 46,540, 31,941, 30,319 unigenes of *B. japonicum* were annotated into NR, Swiss-Prot, GO database (**Table 1**). For genomic sequences corrected, their CDS sequences are fully identical to their corresponding proteins, ensuring reliability of subsequent analysis. The summary statistics of gene correction are shown in **Supplementary Table S1**.

### Amphioxus Evolution Rate and Molecular Phylogeny

**FIGURE 2:**
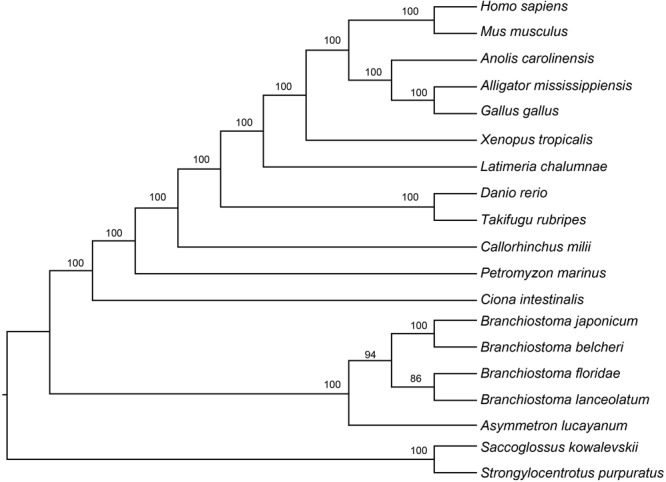
Results of species tree analyses from 19-species-dataset. Branch support was estimated by computing the local posterior probability.

To further clarify the phylogenic relationships among amphioxus, we performed a phylogenetic analysis with five species of cephalochordates (*B. belcheri, B. japonicum, B. lanceolatum, B. floridae*, and *A. lucayanum*), 11 vertebrate species (human, mouse, American alligator, green lizard, chicken, western clawed frog, coelacanth, fugu, zebrafish, elephant shark, and lamprey), one urochordate (sea squirt), and two outgroups (acorn worm and sea urchin). A 19-way concatenated multiple protein alignment, based on 397 orthologous gene groups intersected by HaMSTR and BRH methods (442 for HaMSTR and 415 for BRH), was used to construct the phylogenetic tree. Furthermore, the branch length (the expected amino acid substitution rate) was calculated for each species based on ML (**Figure 1B**), BI (**Supplementary Figure S1**), and PhyloBayes (**Supplementary Figure S2**). These three inference methods obtain congruent tree topologies with nearly identical branch lengths (**Figure 1B**); nodal support values are generally high (PP = 0.95–1.0, BS = 86–100); moreover, these values are stronger in the BI tree than in the ML tree, as has been previously reported (Yuan et al., 2015; Yuan et al., 2016). The species tree estimated by ASTRAL-II is very similar to the tree estimated in the concatenated analysis, with most nodes showing 100% the local posterior probability (**Figure 2**). Regardless of which analyzed method (ML, BI, or PhyloBayes) was used, the branch lengths of all amphioxus except for *B. lanceolatum* were consistently shorter than those of vertebrates, even shorter than that of the elephant shark (*Callorhinchus milii*) as the slowest evolving vertebrate (Venkatesh et al., 2014). The sea squirt showed the longest branch length in the phylogenetic tree. Interestingly, the branch length of *B. lanceolatum* is longer than those of other amphioxus and vertebrates investigated in this study. Based on this difference in branch length, the amino acid substitution rate of *B. lanceolatum* was assessed to be ∼1.3 times higher than that of fugu (*Takifugu rubripes*) with the fastest evolutionary rate among vertebrates. We conducted Tajima’s relative rate test (Tajima, 1993) based directly on the concatenated protein sequence alignment of pairwise tests between *B. lanceolatum* and fugu, which demonstrated a significant result (*P-*value below 0.05). In addition, the phylogenetic trees consistently support a phylogeny of [(*B. belcheri* + *B. japonicum*) + (*B. lanceolatum* + *B. floridae*) + *A. lucayanum*), regardless of the utilized analytical method.

### Estimates of Divergence Time Among Amphioxus Species

**FIGURE 3:**
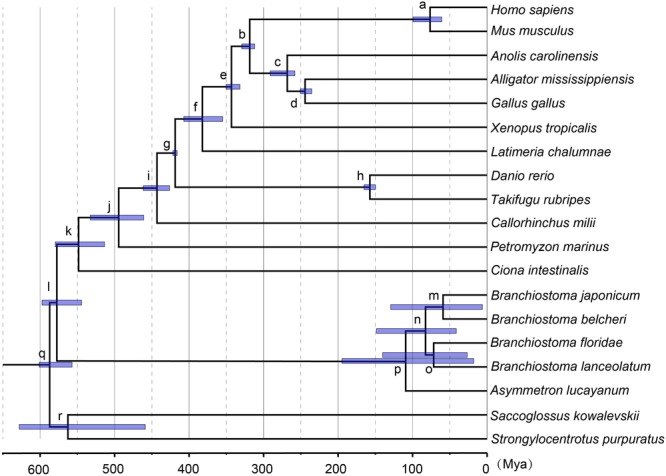
Estimates of the divergence time frame of cephalochordate evolution using MCMCTree. The lower-case letters above the lilac bar represent internal node labels (nodes a–r). All corresponding time constraints, mean divergence times, and 95% confidence intervals (CI) obtained by the Bayesian estimations are tabulated in **Table 2**. Each lilac bar represents the 95% CI of the corresponding estimate.

Based on the ML tree, we performed MCMCTree analysis for the divergence times among all 19 tested species (**Figure 3**). Eleven time constraints were used across the phylogenetic tree (see section “Materials and Methods”). The Deuterostomia divergence times (root age) were employed as 532.67–598.27 and 585.50–698.50 Mya, respectively, based on previous investigations (Yue et al., 2014). The results for all age estimates with 95% CI (confidence interval) and mean values are shown in **Tables 2, 3**. Regardless of which root age we used, the most recent common ancestor of the living amphioxus species (the divergence time between *Asymmetron* and *Branchiostoma*) was estimated to be ∼104 Mya; the early splits within each main clade occurred at ∼87 Mya; *B. lanceolatum* diverged from *B. floridae* at ∼72 Mya, and the divergence time between *B. belcheri* and *B. japonicum* was ∼61 Mya. In addition, to ensure the robustness of estimation results in the divergence time frame, R8S analysis was employed using the same set of time constraints (with a root age of 532.67–598.27 Mya) that were used for the MCMCTree estimation. All divergence results with 95% CIs intervals and mean values are shown in **Table 2**. Despite the obviously different divergence times in several nodes, e.g., f, k, q, and r nodes, the r8s analyses overall supported the MCMCTree results, particularly within the cephalochordate. Consequently, the divergence times calculated with the MCMCTree were reliable and were directly used for the further discussion.

**Table 3:** Bayesian MCMC estimations for the divergence times of each internal node shown in **Figure 2**, assuming that the Deuterostomia divergence (root calibration constraints) occurred at [585.50–698.50] Mya.

		Calibration
		constraints	MCMCTREE	
Node			Mean
index	Node name	[Min, Max] (Mya)	(Mya)	95% CI (Mya)
a	Eutheria	[61.50, 100.50]	73.34	[60.85–98.57]
b	Amniote	[312.30, 330.40]	318.57	[311.92–329.16]
c	Ankylopoda	[259.70, 299.80]	267.54	[257.93–290.14]
d	Archosauriformes	[235.00, 250.40]	244.41	[235.51–250.75]
e	Tetrapoda	[330.40, 350.10]	343.41	[332.18–350.56]
f	Sarcopterygii	–	382.33	[356.45–406.58]
g	Osteichthyes	[416.00, 421.75]	418.74	[416.16–421.72]
h	Acanthopterygii	[149.85, 165.20]	157.37	[149.85–165.11]
i	Gnathostomata	[412.75, 462.50]	447.07	[428.73–462.66]
j	Vertebreta	[460.60, –]	530.01	[477.45–592.12]
k	Olfactores	[520.00, –]	616.81	[554.53–667.15]
l	Chordata	–	662.19	[603.55–697.00]
m	–	–	60.93	[10.21–136.41]
n	Branchiostomidae	–	84.92	[41.02–162.19]
o	–	–	71.71	[31.93–149.68]
p	Cephalochordata	–	100.11	[23.47–211.63]
q	Deuterostomia	[585.50–698.50]	677.64	[628.58–709.37]
r	Ambulacraria	–	601.93	[478.94–712.88]

### Identification and Functional Analysis of Shared and Species-Specific Protein Domains Among Amphioxus Species

Domains are independent functional evolutionary units that can be independently folded (Vogel et al., 2004); most of them have ancient original history (Itoh et al., 2007). To investigate the function of ancient and species-specific domains among amphioxus, we firstly retrieved 10,106, 10,708, 8,912, 8,313, and 9,821 domains from *A. lucayanum, B. belcheri, B. floridae, B. lanceolatum*, and *B. japonicum*, respectively, by searching for homologous domains in the Pfam-A database. Among these domains, 4,814 (group 1, G1) were shared between the genera *Asymmetron* and *Branchiostoma* (**Supplementary Figure S3**) and were retained and handed down from their common ancestor.

Furthermore, gene ontology (GO) enrichment analysis for G1 found that 162 enriched GO terms belonging to the “biological process” subcategory are primarily related to development, cellular process and function, and metabolic processes (**Supplementary Table S2**). Furthermore, we detected 492 (group 2, G2), 361 (group 3, G3), 427 (group 4, G4), 255 (group 5, G5), and 366 (group 6, G6) species-specific domains in *B. belcheri, B. floridae, B. lanceolatum, B. japonicum*, and *A. lucayanum*, respectively (**Supplementary Tables S3–S7**). To examine whether these species-specific domains had biological functionality, we also investigated the enrichment of GO terms for those in each of G2-6 (**Supplementary Table S8**). Among 21 enriched GO terms in G2 (*B. belcheri*), those associated with the immune, inflammatory, stimulus response, apoptosis, and phagocytosis stood out in particular. For G3 (*B. floridae*), 42 GO terms were enriched, and those associated with development, immunity, and apoptosis were overrepresented. Next, 33 GO terms enriched from G4 (*B. lanceolatum*) were primarily involved in lipid storage and regulation, apoptosis, differentiation and movement of immune cells, and response to stimuli. In G5 (*B. japonicum*), 18 enriched GO terms were primarily related to lipoprotein oxidation and metabolism, antioxidant motions, apoptosis, and immune response. The 37 GO terms enriched in G6 (*A. lucayanum*) were involved in organics and energy metabolism, muscle development, and immunity system process. In general, GO terms involving innate immunity and apoptosis were most commonly enriched by these species-specific domains, follow by GO terms related to lipid metabolism and regulation as well as tissue development, suggesting a central role of these related domains in amphioxus speciation.

## Discussion

This study analyzed large-scale omics data of multiple amphioxus species (>2 species) to estimate their molecular phylogeny. Our phylogenomic analyses, based on the supergene dataset with four analytical methods, including three gene trees and one species tree, obtained a highly supported phylogeny of [(*B. belcheri* + *B. japonicum*) + (*B. lanceolatum* + *B. floridae*) + *A. lucayanum*]. This result was consistent with previous analyses that were based on the 12S rRNA (Zhong et al., 2009) and the whole sequences of mtDNA (Kon et al., 2007); however, they were incongruent to the results obtained with 13 PCGs, which indicated a phylogenetic relationship of ((((*B. belcheri* +*B. japonicum*) + *B. lanceolatum*) + *B. floridae*) + *A. lucayanum*). This discrepancy is likely caused by varying evolutionary and/or selection pressures, as has been reported for other animals (Havird and Santos, 2014; Yuan et al., 2018). Several studies indicated that the phylogenies obtained from transcriptomic, genomic, or complete mtDNA represent the highest phylogenetic performance and allow for better results than phylogenies from single gene and small-scale gene sets (Philippe et al., 2005; Yuan et al., 2018). Therefore, the obtained results indicated *B. lanceolatum* as a sister group of *B. floridae*, rather than (*B. belcheri* + *B. japonicum*). These analyses further indicated that 12S rRNA could generate a phylogeny similar to the massive nuclear genes and whole mitogenome, which may thus be used as a potential molecular marker in the phylogeny research of amphioxus. Certainly, this suggestion needs to be further investigated with more amphioxus species. In addition, Yue et al. (2014) reported a slower evolutionary rate of *B. floridae* and *A. lucayanum*, even compared to the slowest evolving vertebrate known (the elephant shark), and the authors speculated that *Asymmetron* and *Branchiostoma* genera should also be included. Indeed, the newly added amphioxus species *B. belcheri* and *B. japonicum* also showed an extremely slow evolutionary rate, supporting the speculation of Yue et al. (2014). However, we found that *B. lanceolatum* is rapidly evolving, even beyond the level of the fastest evolving vertebrate. *B. lanceolatum* (named the Mediterranean amphioxus) is mainly found at the coast of the largest intercontinental sea (the Mediterranean Sea) and in a continental sea (the Black Sea). Due to these relatively closed environments, their terrestrial discharge and unique climate, both regions (particularly the Mediterranean) show exceptionally high biology diversity (Meynard et al., 2012). Therefore, a relatively closed habitat may be a key factor for driving the rapid evolution of *B. lanceolatum* propelled by its weaker ability to buffer against changing ecological factors. Seawater, warmed by a nuclear power plant since 1980 has been reported to cause rapid evolution of parasite resistance in the European perch of the nearly closed Baltic Sea (Mateosgonzalez et al., 2015). Additionally, one of the longer branches in the phylogenetic tree is the branch connecting the amphioxus to the other clades. Near to this branch are several of the other longest branches in the phylogeny. This pattern seems to be caused by long-branch attraction (LBA). Nevertheless, four methods used for gene and species tree establishment were employed for construction of phylogenetic tree. Particularly, ML analysis method presented relatively lower possibility to generate LBA (Li et al., 2007). Concerning the posterior consensus tree, due to Bayesian implementation of site-heterogeneous codon models, CAT-GTR in PhyloBayes seems to be significantly more robust against LBA, compared to all other models (Lartillot et al., 2013). Furthermore, phylogenetic position of the longer branches was mostly consistent with that reported previously (Huang et al., 2014; Yue et al., 2014). Our analysis thus has been avoided LBA as much as possible. Certainly, more comprehensive analysis was required to further reduce LBA possibility in future studies.

**FIGURE 4:**
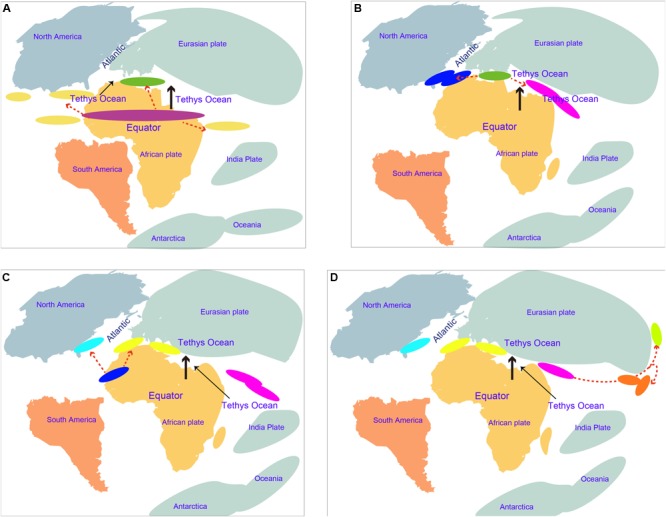
Speciation hypotheses of cephalochordate during Cretaceous and early Cenozoic. **(A)** The African plate moved northward around 104 Mya; the purple oval indicates the common ancestor of existing cephalochordate; the dark green oval indicates the common ancestor of *Branchiostoma*; dark yellow ovals indicate *Asymmetron* ancestors; red dotted arrows indicate divergence and speciation. **(B)** The Africa plate moved northward around 86 Mya; dark blue ovals indicate western groups formed by divergence of the *Branchiostoma* ancestor in the Tethys region between the Eurasian and African plates; pink ovals indicate eastern groups. Continental movement that led to collisions caused the Tethys to shrink further. **(C)** The Atlantic Ocean is widened around 72 Mya; blue ovals indicate *B. floridae* lineage; yellow ovals indicate *B. lanceolatum*. **(D)** Divergence of *B. belcheri* and *B. japonicum* occurred at around 61 Mya; red ovals indicate *B. belcheri* lineage; pale green ovals indicate *B. japonicum*; Eastern groups moved eastward along with the southern margin of the Eurasian plates and speciated in this process.

The estimation of divergence time is very helpful to understand the speciation event and evolutionary history of amphioxus. The obtained molecular dating results can be substantially influenced by a number of factors, e.g., evolutionary models, methods of analysis, and time constraints (Baele et al., 2012; Yuan et al., 2018). This study used multiple methods to estimate the divergence time of amphioxus to avoid the bias of using a single method of analysis only. Additionally, many fossil calibrations (reach 10) and a secondary constraint were used to improve the achieved accuracy of molecular dating. Our age estimation for the main vertebrate groups (slightly older) and the divergence of *Branchiostoma*–*Asymmetron* (slightly younger) was mostly consistent with that reported by Yue et al. (2014), indicating that our obtained results were robust. Five species of amphioxus showed speciation of no more than ∼47 Mya (nodes p to m), indicating that even though most amphioxus species showed a slow evolutionary rate (Putnam et al., 2008; Yue et al., 2014), their speciation process is not as slow as could have been expected, and even exceeds that of most vertebrate groups. Despite the transcriptome of *Epigonichthys*, the third genus of cephalochordates, still being absent, analysis of the complete mtDNA showed a phylogeny relationship of {vertebrates + [*Asymmetron* + (*Branchiostoma* + *Epigonichthys*)]}, demonstrating that *Asymmetron* diverged earlier than the other two genera in cephalochordates (Kon et al., 2007). If mass sequences of *Epigonichthys* were added to our analyses, the first divergence time (∼104 Mya) of living cephalochordates will not become earlier than the current result. Before and at this time, the expansion of the mid-oceanic ridge was driving northward of the African plate according to the Wegener hypothesis of continental drift (Smith, 2014). The movement divided the common ancestors of *Asymmetron* and *Branchiostoma* into multiple isolated geographical populations. Part of these remained at their home areas along the equator and evolved into the *Asymmetron* ancestor, while others were pushed to a higher altitude and evolved into the *Branchiostoma* ancestor in the Tethys region between the Eurasian and African plates (**Figure 4A**). The *Asymmetron* distribution primarily along the equator and low latitudes, showing high similarity with their current distribution pattern (Kon et al., 2006). This further supports the proposed divergence hypothesis between *Asymmetron* and *Branchiostoma*. Furthermore, it is reasonable to speculate that the common ancestor of living cephalochordates likely inhabited areas along the equator and low latitudes. With the northward movement of the African plate toward the Eurasian plate, the *Branchiostoma* ancestors divided into both eastern and western groups in this Tethys region (**Figure 4B**). Notably, due to the reliable gene communication caused by the largely incomplete closure of the Tethys during this period, we suggest that speciation between both groups remained unfinished until ∼87 Mya. At about 110–65 Myr, the Atlantic Ocean gradually opened due to the gradual mid-Cretaceous separation of North America from the Eurasian plate and the breakup of Gondwana (namely Africa and South America). The spread of the Atlantic Ocean constitutes an oceanic barrier due to the enormous depth and width between Europe and the Americas (Frisch and Dawes, 2014; Hou and Li, 2017). Therefore, it is likely that western groups diverged into *B. floridae* at the west coast and *B. lanceolatum* at the east coast of the Atlantic Ocean at ∼72 Mya due to the expansion of the Atlantic Ocean (**Figure 4C**). Similar suggestions have been reported for mollusks and crustaceans (Hou and Li, 2017). India moved northward from its original position and collided with Asia during the Early Eocene (∼50 Mya) (Hou and Li, 2017). A wide eastern region of the Tethys thus remained to be opened ∼61 Mya (Hou and Li, 2017), and was not divided into the Indian Ocean and the Pacific Ocean. We therefore speculate that the original eastern groups in the Tethys region between the Eurasian and African plates moved east through the region between the Eurasian and Indian plates. Subsequently, part of this moving population likely moved into higher latitudes along the eastern coastline of the Eurasian plate, and speciation of *B. belcheri* and *B. japonicum* happened at ∼61 Mya during this period (**Figure 4D**). It is likely that divergence of both species was driven by changes of the habitat caused by this difference in latitude. We thus propose that the oceanic evolution driven by platonic movement supplied an important factor for the divergence and speciation of cephalochordates. Consequently, the distribution pattern of living cephalochordates has a close evolutionary relationship with the tectonic structure.

Comparative domain analysis among different amphioxus species showed no significant difference of the number of domains identified by the whole genomes and transcriptomes. This indicates that domain types (not quantity) of the species were fully obtained through the transcriptome of adult amphioxus. Via GO enrichment analyses, we found that species-specific domains of amphioxus are consistently related to immune response and apoptosis. This result indicates exposure to pathogens in the seawater as a primarily driving force for the origin and evolution of amphioxus domains. This may be furthered by their burying in seafloor sand and relatively poor swimming ability, leading to an accumulation of pathogenic microorganisms. Previous studies reported that rapidly evolving genes between different genera or species of amphioxus were primarily involved in innate immunity (Yue et al., 2014; Zhang et al., 2018). This suggests that rapid evolution in sequence played a key role in the evolutionary origin of species-specific domains. Several species of amphioxus possess special capabilities for lipid utilization, particularly^3^ *B. lanceolatum* (the website of the *B. lanceolatum* genome sequencing project). Our investigations found several GO terms involving lipid storage, metabolism, and regulation in *B. lanceolatum* and *B. japonicum*, perhaps indicating both species as ideal model species for research on the lipid biology of amphioxus. Moreover, this result also indicates species-specific adjustment of energy utilization and production as an important evolutionary step in the speciation history of amphioxus. The common primitive domains of cephalochordates primarily participated in development, cellular process and function, and metabolic process to ensure normal basic biological processes.

In summary, this study represents the first large-scale phylogenomic analysis including most major cephalochordate genera based on transcriptomic data. A phylogeny of [(*B. belcheri* + *B. japonicum*) + (*B. lanceolatum* + *B. floridae*) +*A. lucayanum*], clarifying the phylogenetic position of *B. lanceolatum*. Moreover, we proposed that 12S RNA reported previously mitochondrial genes may be a reliable molecular marker in phylogenetic analyses of cephalochordates. We firstly proposed that the currently living amphioxus species were not an old group, and most likely radiated during the Cretaceous, and their diversification and speciation was driven by primarily the platonic movements during this period. In addition, a batch of species-specific and ancestral protein domains was identified and functionally analyzed among cephalochordates using bioinformatics, indicating several factors that likely promoted the adaptation of amphioxus species to their respective habitats. Our study adds to the understanding of the cephalochordate evolutionary relationships and provides insight into their divergence history and speciation characteristics.

## Author Contributions

L-BL, J-YC, and Q-LZ conceived and designed the experiments. Q-LZ, G-LZ, M-LY, Z-XD, and H-WL performed the experiments. Q-LZ, M-LY, JG, FW, and X-YD analyzed the data. Q-LZ, L-BL, and J-YC wrote the paper. L-BL, M-LY, and J-YC revised the manuscript.

## Conflict of Interest Statement

The authors declare that the research was conducted in the absence of any commercial or financial relationships that could be construed as a potential conflict of interest.

## Footnotes

1 http://www.hmmer.org/

2 https://cran.r-project.org/

3 http://www.genoscope.cns.fr/spip/L-espece-d-amphioxus-un-modele.html

## Supplementary Material

FIGURE S1 19-way Bayesian phylogenetic tree inferred from a concatenated orthologous gene matrix. Numbers on branches represent Bayesian posterior probabilities.

FIGURE S2 19-way PhyloBayes phylogenetic tree inferred from a concatenated orthologous gene matrix.

FIGURE S3 Domain venn diagram of five amphioxus species.

TABLE S1 Summary statistics of sequence correction.

TABLE S2 List of GO terms enriched by genes containing domains that are shared among all tested amphioxus.

TABLE S3 Information of lineage-specific domains in *Branchiostoma belcheri*.

TABLE S4 Information of lineage-specific domains in *Branchiostoma floridae*.

TABLE S5 Information of lineage-specific domains in *Branchiostoma lanceolatum*.

TABLE S6 Information of lineage-specific domains in *Branchiostoma japonicum*.

TABLE S7 Information of lineage-specific domains in *Asymmetron lucayanum*.

TABLE S8 List of GO terms enriched by genes containing lineage-specific domains in each species of amphioxus.
