# Supplementary figures and images for: A Phylogenomic Framework and Divergence History of Cephalochordata Amphioxus
Source: Front Physiol. 2018 Dec 18;9:1833. doi: 10.3389/fphys.2018.01833 (PMC6305399; doi:10.3389/fphys.2018.01833)

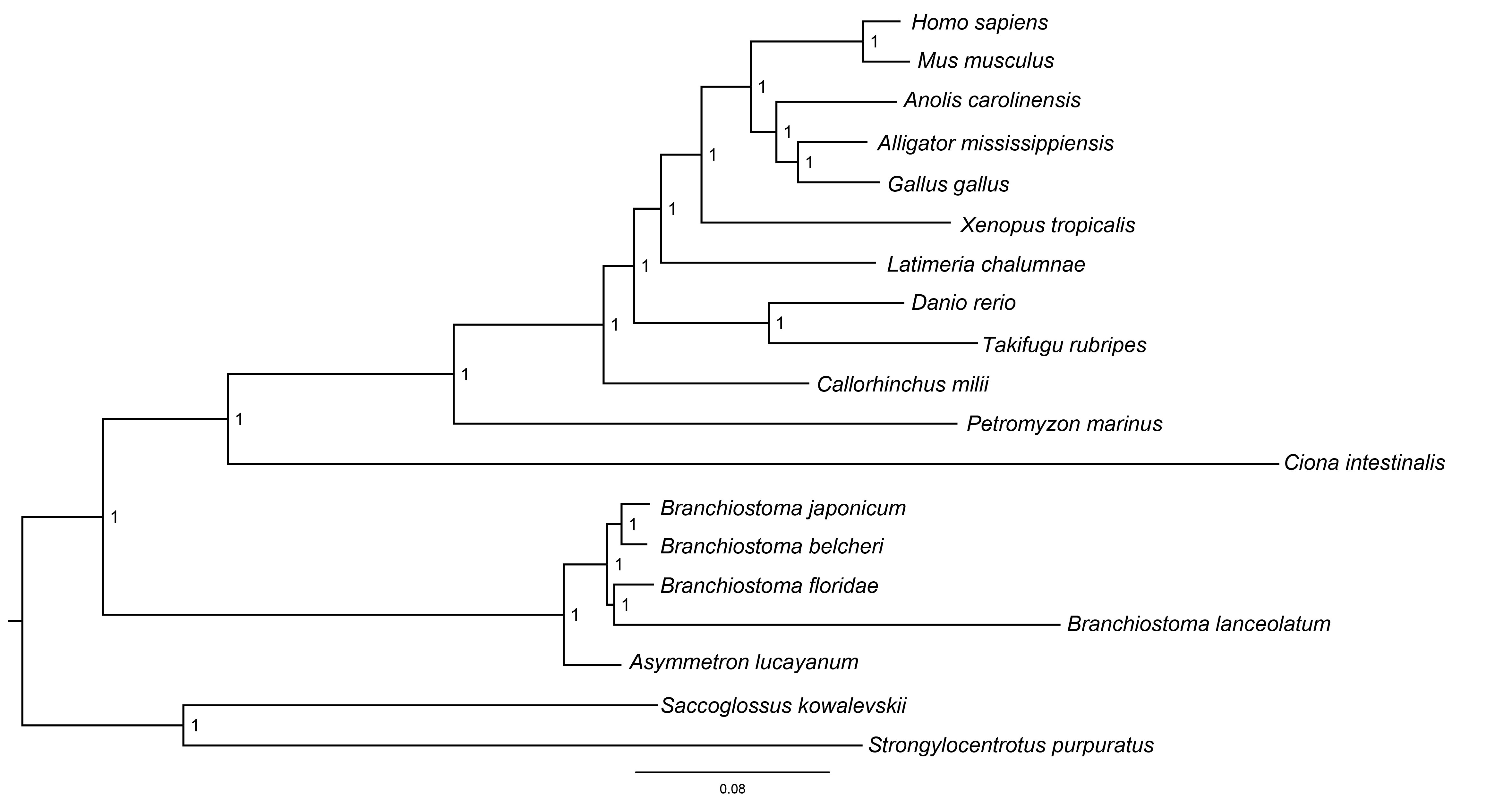

Supplement: FIGURE S1 — 19-way Bayesian phylogenetic tree inferred from a concatenated orthologous gene matrix. Numbers on branches represent Bayesian posterior probabilities. [file Image_1.JPEG]

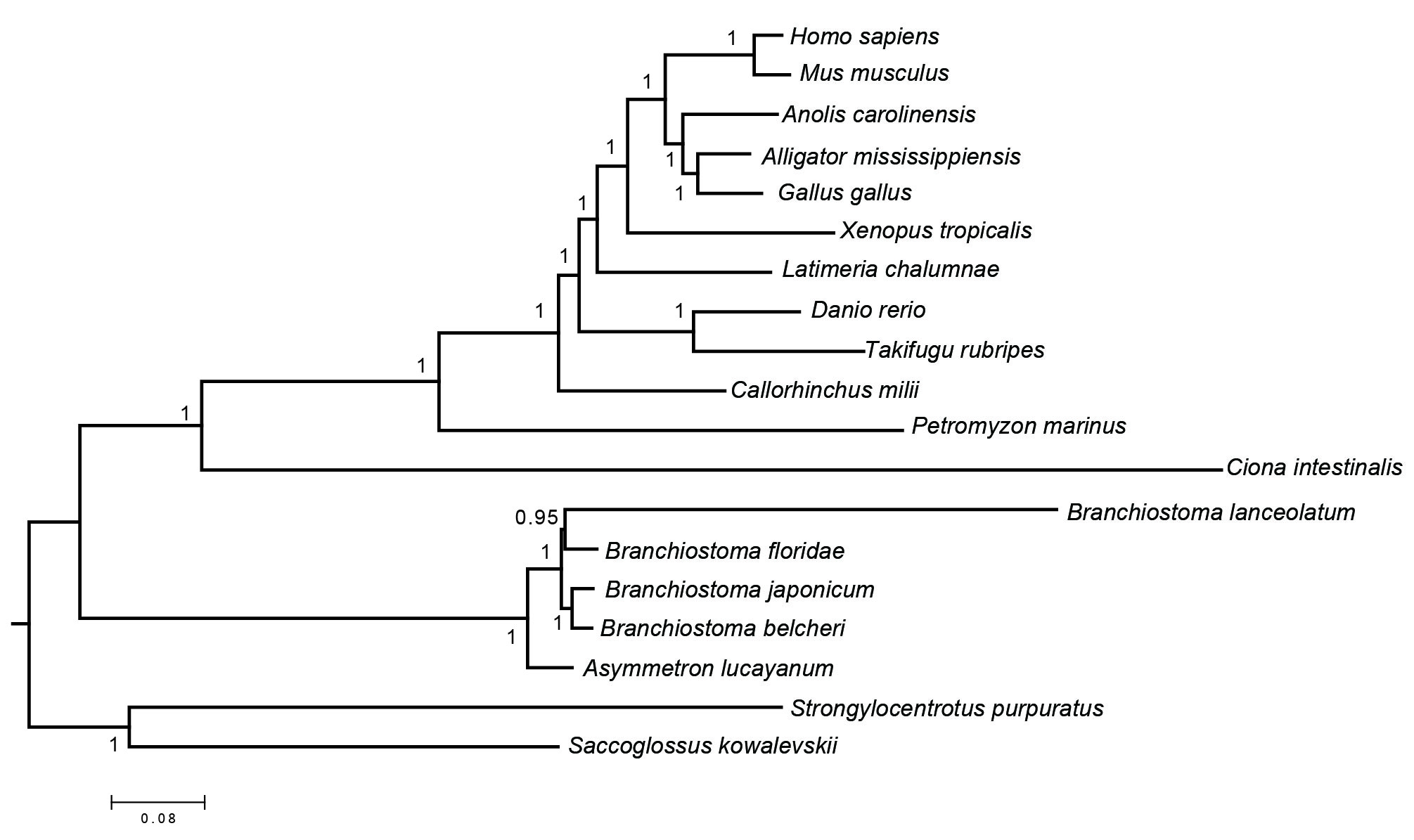

Supplement: FIGURE S2 — 19-way PhyloBayes phylogenetic tree inferred from a concatenated orthologous gene matrix. [file Image_2.JPEG]

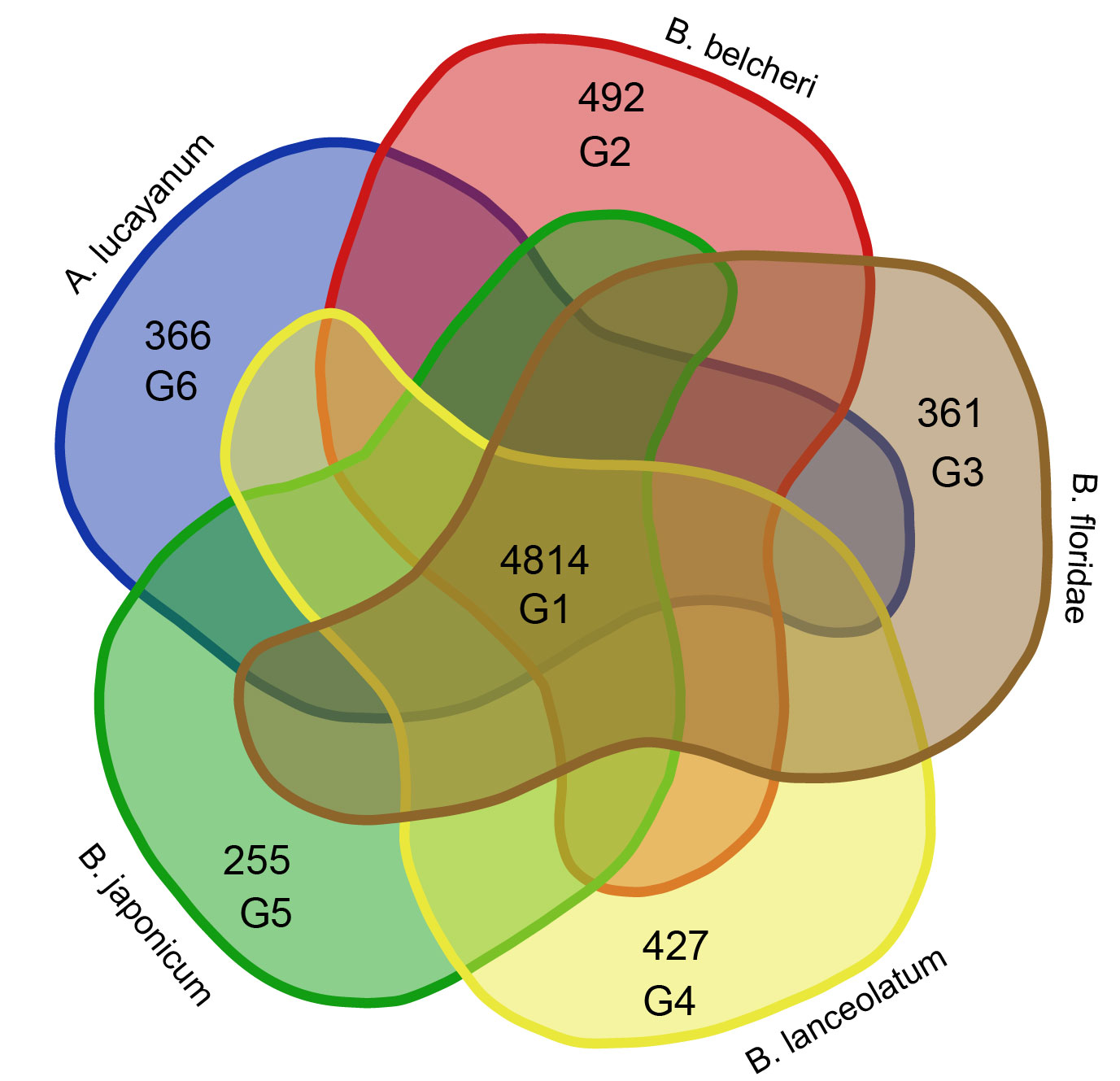

Supplement: FIGURE S3 — Domain venn diagram of five amphioxus species. [file Image_3.JPEG]
